# Smoking patients with laryngeal cancer screened with a novel immunogenomics-based prognostic signature

**DOI:** 10.3389/fgene.2022.961764

**Published:** 2022-07-14

**Authors:** Yujie Shen, Han Zhou, Shikun Dong, Weida Dong, Liqing Zhang

**Affiliations:** ^1^ Department of Otorhinolaryngology Head and Neck Surgery, Eye, Ear, Nose and Throat Hospital, Fudan University, Shanghai, China; ^2^ Department of Otorhinolaryngology, The First Affiliated Hospital of Nanjing Medical University, Nanjing, China; ^3^ Department of Otolaryngology Head and Neck Surgery, Zhongda Hospital, Southeast University, Nanjing, China

**Keywords:** Lscc, prognosis, signature, outcome, TCGA

## Abstract

The immune system greatly affects the prognosis of various malignancies. Studies on differentially expressed immune-related genes (IRGs) in the immune microenvironment of laryngeal squamous cell carcinoma (LSCC) have rarely been reported. In this paper, the prognostic potentials of IRGs were explored in LSCC patients with smoking use. The RNA-seq data containing IRGs and corresponding clinical information of smoking LSCC patients was obtained from The Cancer Genome Atlas (TCGA). Differentially expressed IRGs were identified and functional enrichment analysis was used to reveal the pathway of IRGs. Then, IRGs with prognostic potentials in smoking LSCC patients were screened out by univariate Cox regression analysis. Finally, multivariate Cox regression analysis was conducted to assess the prognostic signature of 5 IRGs after adjustment of clinical factors and patients were classified into two subgroups based on different IRGs expression. The prognostic capacity of the model was verified by another independent cohort from Gene Expression Omnibus (GEO) database. Nomogram including the prognostic signature was established and shown some clinical net benefit. These findings may contribute to the development of potential therapeutic targets and biomarkers for the new-immunotherapy of LSCC patients with smoking use.

## Introduction

Laryngeal cancer, a most common malignant tumors in the head and neck, ranks the 11th among the most common malignant tumors in men (Marioni et al., 2006). Among all pathogenic factors, smoking is one of the most crucial risk factors for laryngeal cancer (Jethwa and Khariwala, 2017). Notably, patients with invasive and metastatic laryngeal cancer have a poor prognosis. More than 95% cases of laryngeal cancer are laryngeal squamous cell carcinoma (LSCC). Despite great strides on LSCC treatment in recent years, the mortality rate of LSCC remains high (Hardisson, 2003). Due to the lack of symptoms, LSCC in the early stage is often neglected. Furthermore, because of the screening and diagnostic limitations, the detective rate of LSCC, especially early-stage LSCC, is relatively low. Therefore, identifying effective biomarkers and target genes is of vital importance in improving diagnostic and therapeutic efficacies for LSCC (Suh et al., 2014).

Previous studies have confirmed the significance of tumor immunoreaction in the tumor microenvironment. Prognostic signatures based on immune-related genes (IRGs) have been proposed for non-squamous non–small cell lung cancer (Li et al., 2017) and papillary thyroid cancer (Lin et al., 2019). Tumor-infiltrating immune cells with different densities, localizations and types have been identified as prognostic factors in lung cancer (Kadota et al., 2015), colorectal cancer (Dahlin et al., 2011) and breast cancer (Adams et al., 2014). Nevertheless, the clinical relevance and prognostic significance of IRGs in LSCC are yet to be elucidated.

Herein, we aimed to explore potential therapeutic targets and biomarkers for immunotherapy of LSCC patients with smoking use. TCGA datasets containing IRG expression profiles and clinical information of patients were analyzed. An immunogenomics-based prognostic index of smoking LSCC patients was developed and the potential classification capability was further discussed and verified.

## Materials and Methods

### Clinical samples and data acquisition

We downloaded the RNA sequencing (RNA seq) data and corresponding clinical information from The Cancer Genome Atlas (TCGA), the largest cancer gene information database to collect relevant data (Lee, 2016). We collected the transcription profile and clinical information of 112 smoking LSCC patients, which including 10 laryngeal normal samples and 102 tumor samples. The clinical information of TCGA cohort is demonstrated in Supplementary Table S1. At the same time, we screened the NCBI GEO database (www.ncbi.nlm.nih.gov/geo/) and selected the GSE65858 dataset for validation, which including 222 smoking patients with head and neck squamous cell carcinomas (HNSCC). The clinical information of GSE65858 cohort is demonstrated in Supplementary Table S2. We also obtained a list of IRGs (Supplementary Table S3) via the Immunology Database and Analysis Portal (ImmPort) database (https://www.immport.org) (Bhattacharya et al., 2018).

### Differential expressed analysis

We tried to identified differential expressed IRGs in smoking LSCC patients via R software (version: x64 3.2.1) and package “limma” (Silva and Richard, 2016). The screening criteria were: | log(fold change) | ≥ 1 and adj. *p* < 0.05.

### Pathway enrichment analysis

To explore the potential molecular mechanisms of the IRGs, we performed Kyoto Encyclopedia of Genes and Genomes (KEGG) signaling pathway analysis (Kanehisa and Goto, 2000) on these differentially expressed IRGs using package Cluster profiler of R.

### Construction of a prognostic index model of smoking laryngeal squamous cell carcinoma patients based on immune-related genes

Prognosis-related IRGs were filtered out by univariate Cox regression analysis. More, we performed least absolute shrinkage and selection operator (LASSO) analysis to avoid overfitting, and a prognostic index model was constructed based on multivariate Cox regression analysis. The risk score formula of our prognostic signature was as follows:

Risk score = *coef* ∗ *Exp* (gene A) + *coef* ∗ *Exp* (gene B) + *coef*i ∗ *Exp*i (gene i) (Yu et al., 2019)

### Verification of the prognostic capacity based on prognostic signature

Smoking LSCC patients were divided into the high-risk group and the low-risk group based on the median risk score value. Receiver operating characteristic (ROC) analysis were constructed via the package survival ROC in R to validate the performance of prognostic index (Kamarudin et al., 2017). Survival analysis of two groups with a threshold of *p* < 0.05 was carried out by package survival and survminer in R. In addition, we performed principal component analysis (PCA) in order to test the classification ability of the prognostic signature and observed the prognostic value of prognostic index by introducing clinical factors like age and grade. Clinical survival analysis based subgroup was also conducted and a value of *p* < 0.05 was considered significant statistically. Also, the same validation and analysis methods were used in GSE65858 cohort for further external validation.

### Immune cell infiltration and drug sensitivity analysis

Between low- and high-risk groups, CIBERSORT (Kawada et al., 2021) and single-sample Gene Set Enrichment Analysis (ssGSEA) algorithms were used to evaluate the infiltration of immune cells. Moreover, on the basis of genomics of Drug Sensitivity in Cancer (GDSC) database (https://www.cancerrxgene.org/), effective chemotherapeutic drugs targeting specific group were screened out (Yang et al., 2013).

### Independent prognostic analysis and building a predictive nomogram

Univariate and multivariate Cox regression analysis were performed to determine the independent prognostic factors for smoking LSCC patients. A nomogram was then constructed to investigate the likelihood of one-, three- and five-year OS for LSCC. Finally, we plotted time-dependent calibration curves and ROC curves of the nomogram to observe the relationship between the predicted probability of the nomogram and the ideal rate.

### Statistical analysis

All statistical analyses were carried out by using R software (version: x64 3.2.1) and GraphPad Prism 7 software. A *p*-value < 0.05 was regarded as statistically significant.

## Results

### Identification of differential expressed immune-related genes

**FIGURE 1:**
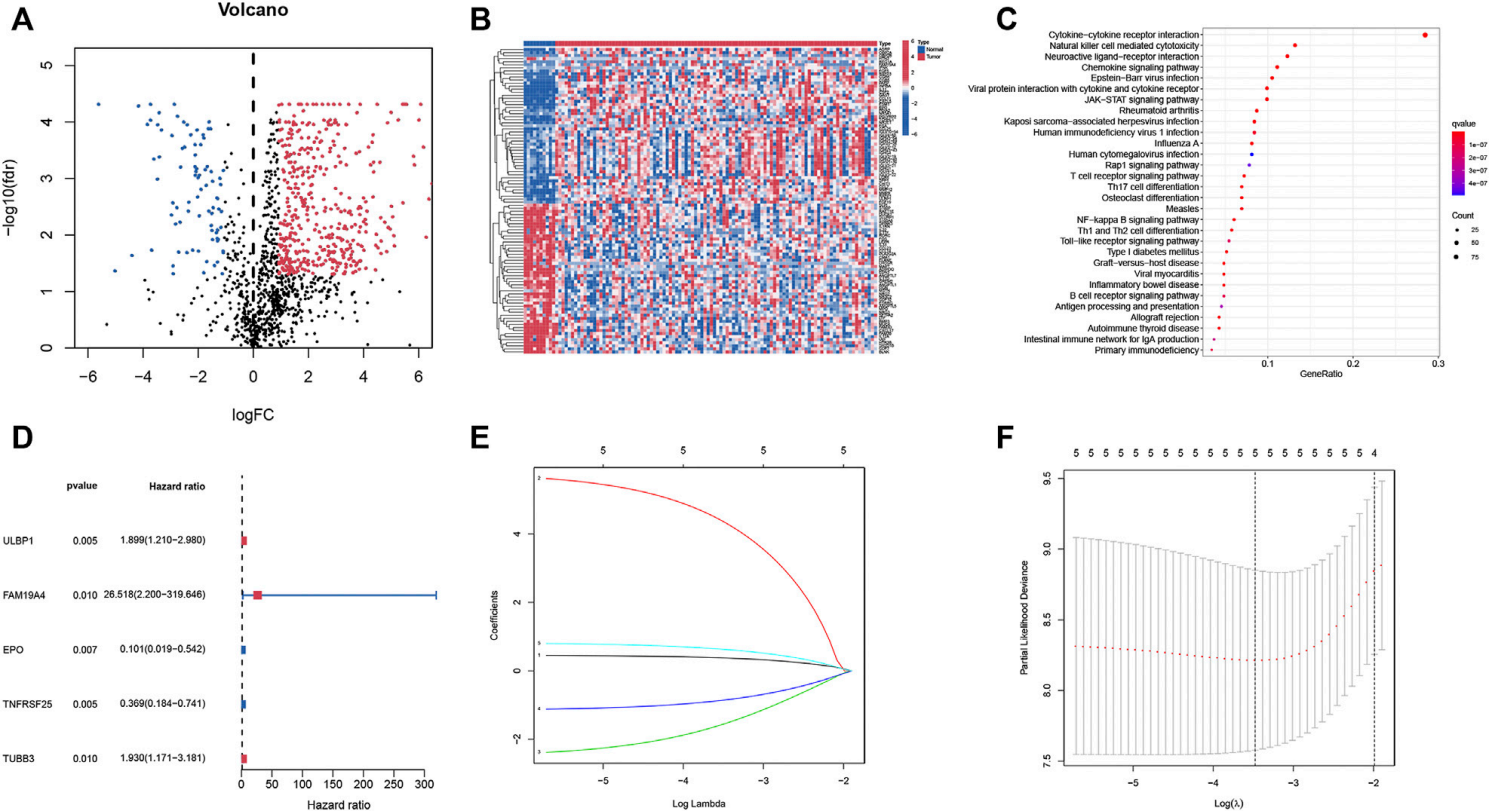
Identification of IRGs and prognosis-related IRGs in smoking LSCC patients. **(A)** Volcano plot of differential expressed IRGs; **(B)** Heatmap of differential expressed IRGs; **(C)** Significant KEGG pathway terms of IRGs; **(D)** Univariate Cox regression analysis of differential expressed IRGs; **(E)** LASSO coefficient profiles; **(F)** LASSO deviance profiles.

522 differential expressed IRGs were identified (Supplementary Table S4), including 437 up-regulated and 85 down-regulated IRGs. Cluster analyses on differential expressed IRGs were presented in Figures 1A,B. Subsequently, the KEGG signaling pathway analysis (Figure 1C) found that these differential expressed IRGs were mainly abundant in cytokine-cytokine receptor interaction, natural killer cell mediated cytotoxicity and neuroactive ligand-receptor interaction.

### Identification of prognosis-related immune-related genes and construction of the prognostic index model.

After integrating clinical information from TCGA and analyzing using the univariate Cox regression, five prognostic IRGs were identified. Forest plots (Figure 1D) showed that two IRGs may be the protective factors of smoking LSCC patients, while the remaining three IRGs could be risk factors of smoking LSCC patients. Taken together, these results suggested that these IRGs may be of significance in the development of LSCC. Then, LASSO regression was performed and selected the five IRGs for constructing a prognostic signature (Figures 1E,F). Based on the results of the multivariate Cox regression analyses, a prognostic index model was constructed. Risk scores of smoking LSCC patients were calculated as follows:

Risk score = ULBP1 expression ∗ 0.376808079833036 + FAM19A4 expression ∗ 4.33210085517556 + EPO expression ∗ -1.54033312666808 + TNFRSF25 expression ∗ -0.85010355482497 + TUBB3 expression ∗ 0.62646678201039.

### Verification the prognostic capacity of the prognostic index model

**FIGURE 2:**
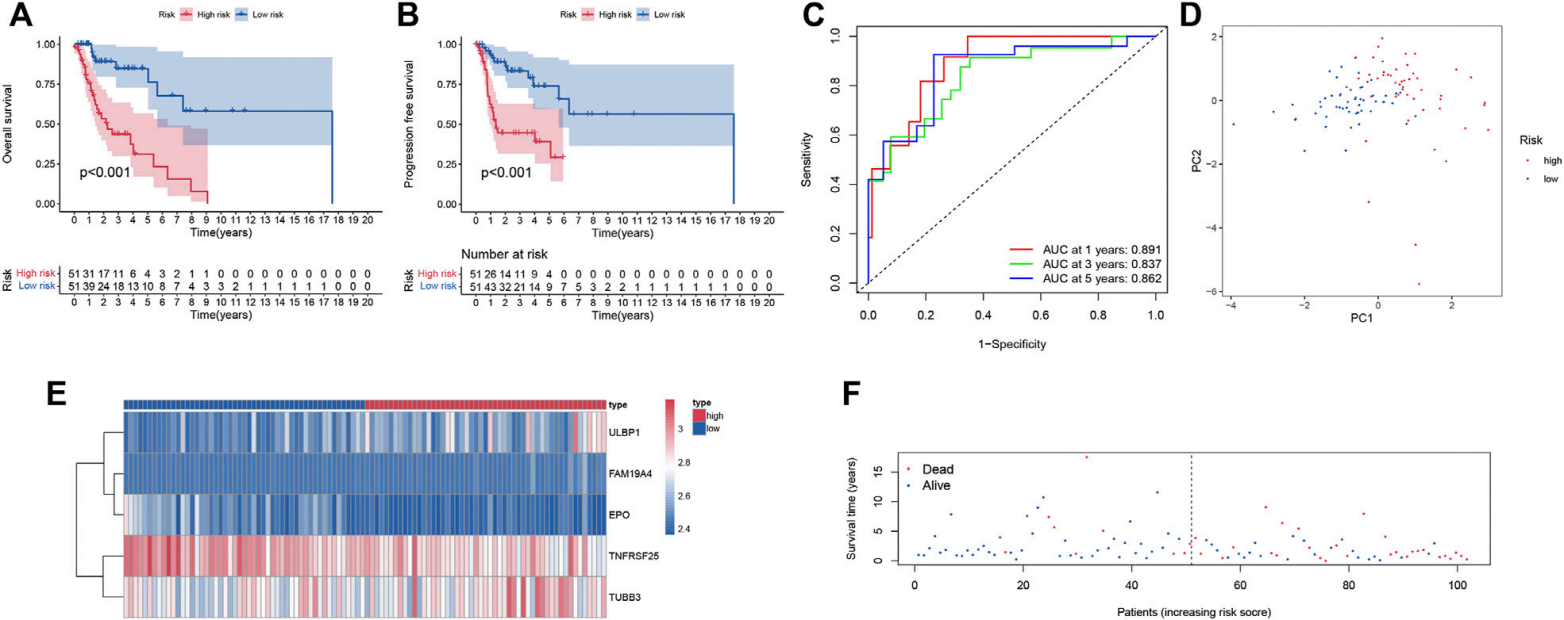
Predictive power of the prognostic signature. **(A)** Kaplan-Meier survival analysis for overall survival; **(B)** Kaplan-Meier survival analysis for progression free survival; **(C)** ROC curve of the prognostic index model; **(D)** Principal component analysis; **(E)** Heatmap of expression profiles of included IRGs; **(F)** Survival status plot of the prognostic index model.

**FIGURE 3:**
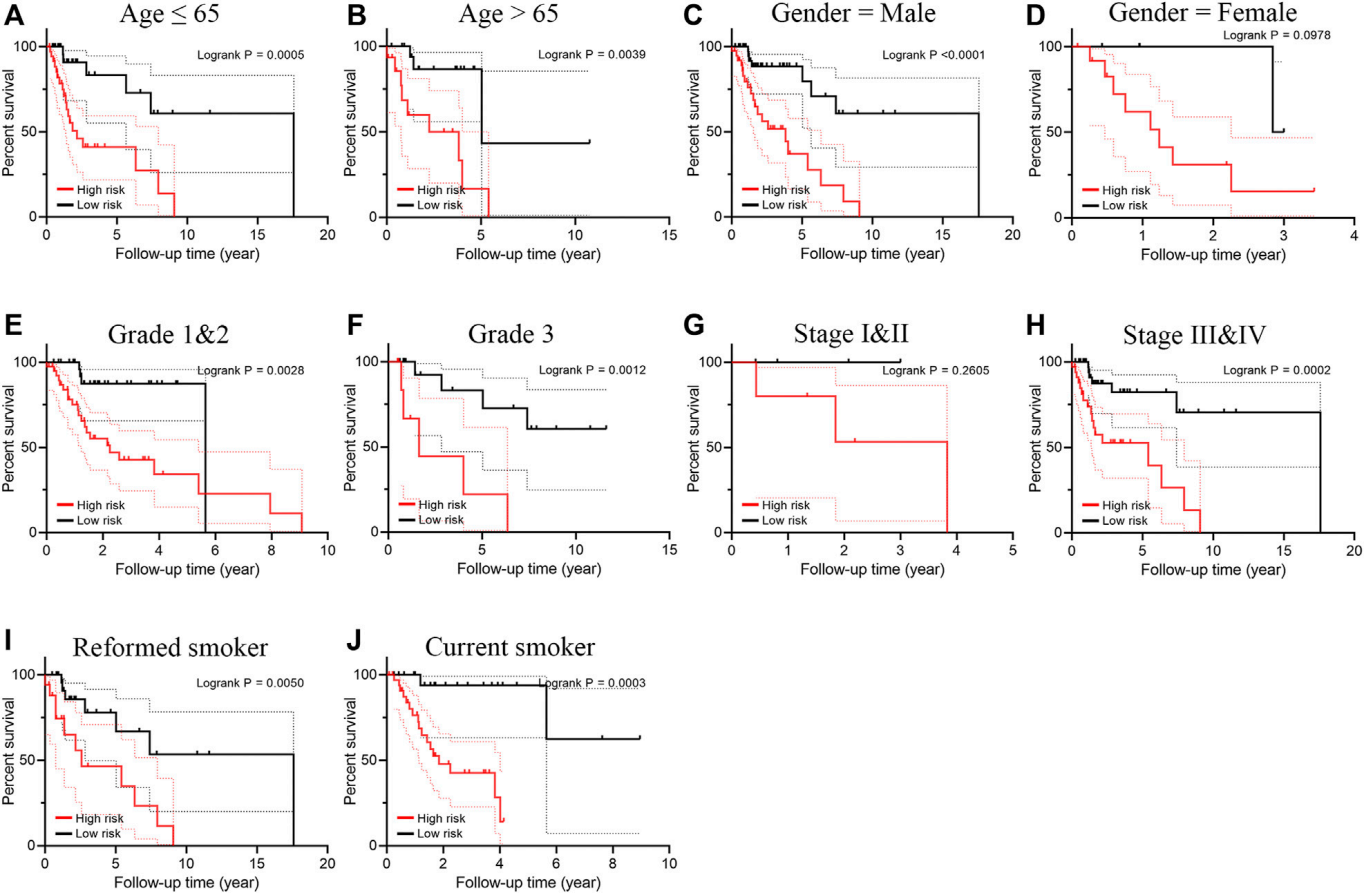
Subgroup survival analysis for smoking patients with LSCC according to the prognostic index stratified by clinical factors. **(A)** Age ≤ 65; **(B)** Age > 65; **(C)** Male; **(D)** Female; **(E)** Grade 1 and 2; **(F)** Grade 3; **(G)** Stage I and II; **(H)** Stage III and IV; **(I)** Reformed smoker; **(J)** Current smoker.

Smoking LSCC patients were separated into the high-risk group and the low-risk group based on the median level of risk score. Survival analysis showed that the overall survival (OS) and progression free survival (PFS) rate in the high‐risk group was remarkably lower than that in the low‐risk group (*p* < 0.001, Figures 2A,B). The area under curve (AUC) value for one-year, three-year, and five-year OS was 0.891, 0.837, and 0.862, respectively (Figure 2C), suggesting a great performance of the prognostic index model in predicting the prognosis of smoking LSCC patients. Moreover, the prognostic index model showed a great clustering ability in PCA plot (Figure 2D). Different expression of the five IRGs and high mortality were observed in high-risk group compared to low-risk group (Figures 2E,F). In order to assess the prognostic capacity of prognostic index more comprehensively, we conducted a stratified analysis of clinical factors. Interestingly, we found that high risk patients in nearly all the subgroups were inclined to have unfavorable overall survival (Figures 3A–J). Similar results (Supplementary Figures S1A–S1E) were observed in another independent cohort (GSE65858 cohort). Of those, the OS in the high-risk group was lower than that in the low-risk group (*p* = 0.166, Supplementary Figure S1A).

### Immune contexts and sensitive drugs between different groups

**FIGURE 4:**
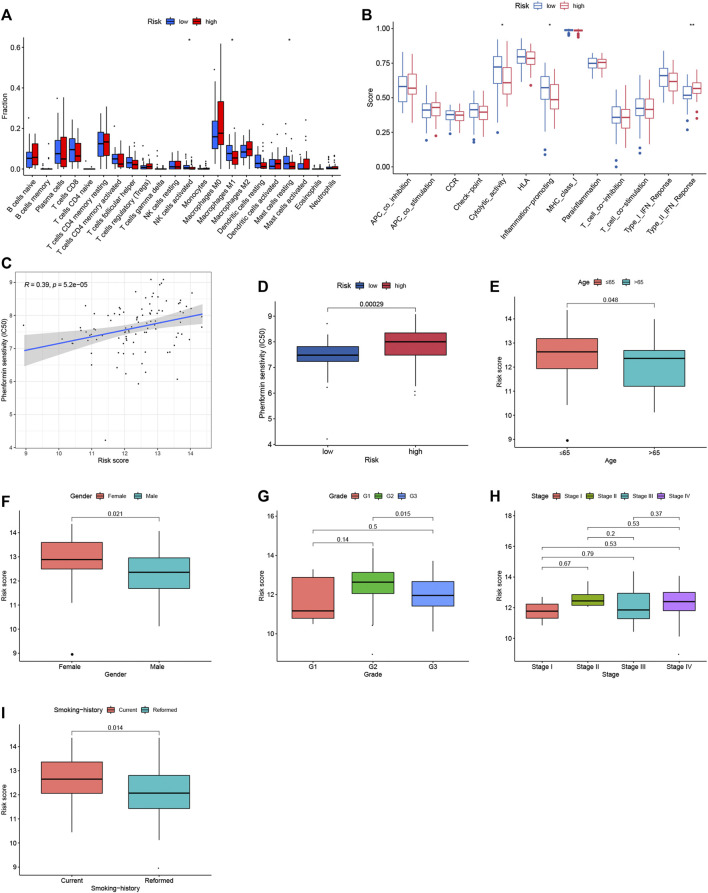
Immune contexts, sensitive drugs and clinical correlations between different groups. **(A)** Relationship between the immune‐related prognostic index and the infiltration abundances of immune cells; **(B)** Immune activities in different groups; **(C)** Correlation analysis between Phenformin and risk score; **(D)** Phenformin senstivity; **(E)** Correlation analysis between age and risk score; **(F)** Correlation analysis between gender and risk score; **(G)** Correlation analysis between grade and risk score; **(H)** Correlation analysis between stage and risk score; **(I)** Correlation analysis between smoking history and risk score.

To explore the relationship between the immunogenomics-based prognostic index model and tumor immune microenvironment, we compared the infiltration of immune cells in different risk groups. Through CIBERSORT algorithm, activated NK cells, M1 macrophages and resting mast cells were highly enriched in low-risk group (*p* < 0.05, Figure 4A), which could account for the better outcomes of low-risk group. When it comes to ssGSEA algorithm, cytolytic activity and inflammation promoting were significantly activated in low-risk group (*p* < 0.05, Figure 4B) when type II IFN response were significantly activated in high-risk group (*p* < 0.01, Figure 4B). Moreover, the potential drug targeting smoking LSCC patients was identified (Figures 4C,D). In addition, the relationship between the risk scores and clinical factors of smoking LSCC patients was explored (Figures 4E–I). Remarkably, in smoking LSCC patients, higher IRGs-related signature expression was observed in current smokers than former smokers (*p* = 0.014, Figure 4I).

### Independent prognostic analysis

**FIGURE 5:**
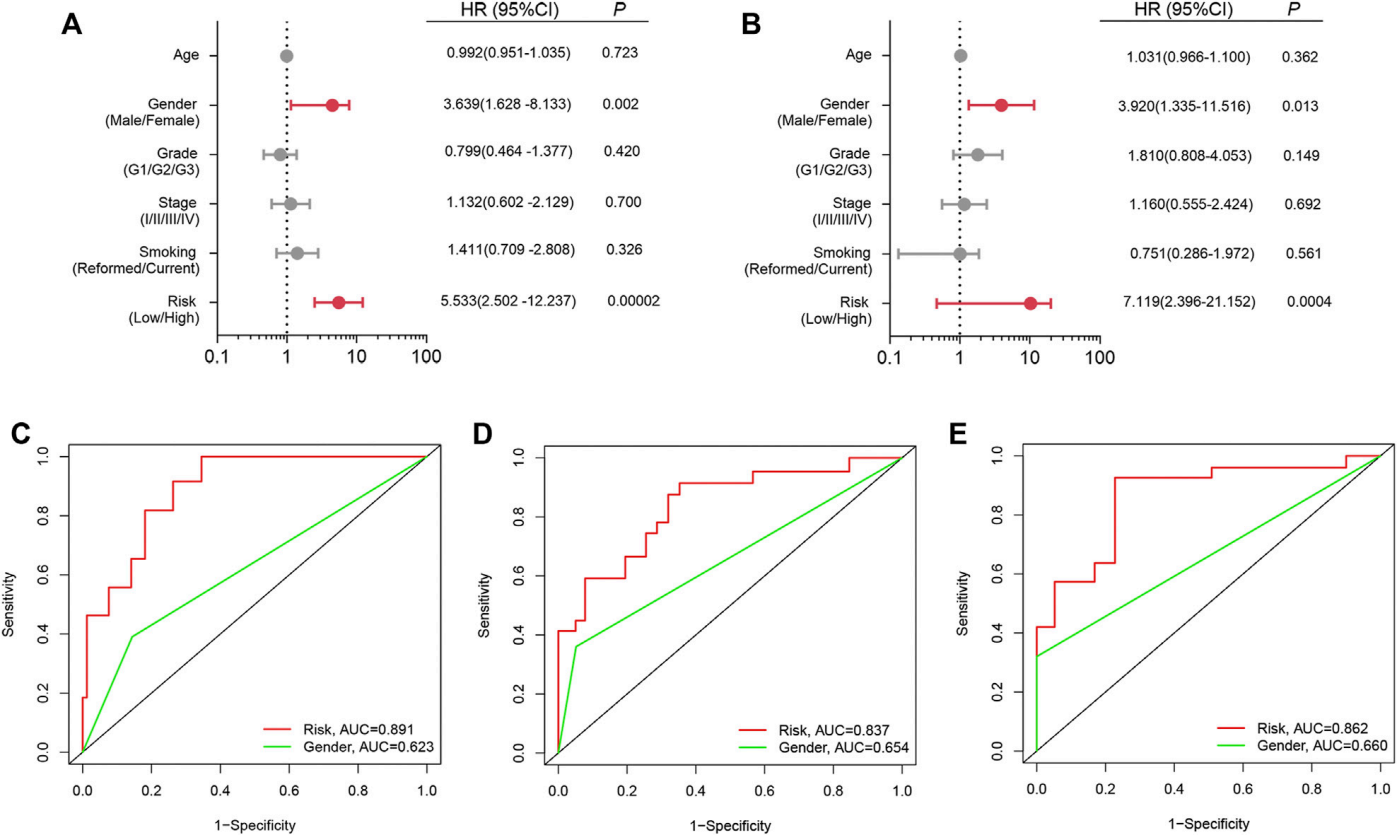
Independent prognostic analysis. **(A)** Univariate analysis; **(B)** Multivariate analysis; **(C)** ROC curve for one-year OS; **(D)** ROC curve for three-year OS; **(E)** ROC curve for five-year OS.

Univariate and multiple regression analyses (Figures 5A,B) suggested that the prognostic signature could become an independent predictor after adjustment of age, gender, tumor grade, tumor stage and smoking status in LSCC patients. Moreover, compared with the other independent predictors, the prognostic index model showed the largest AUC value for one-year, three-year and five-year OS (Figures 5C–E).

### Building and validating a predictive nomogram

**FIGURE 6:**
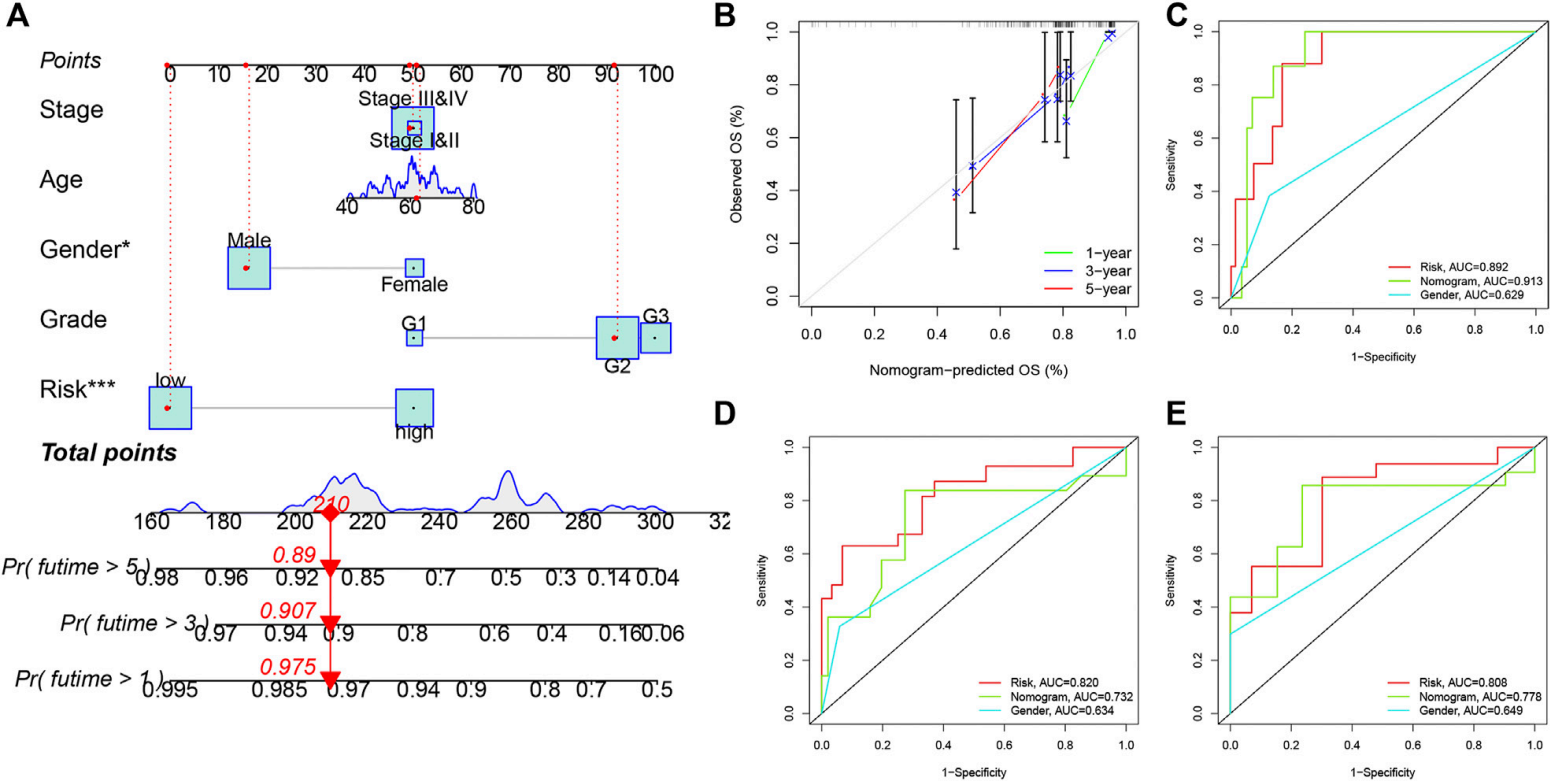
Nomogram for predicting overall survival for smoking LSCC patients. **(A)** The nomogram for predicting the possibility of one-, three- and five-year overall survival of smoking LSCC patients; **(B)** Time-dependent calibration curves of the nomogram; **(C)** ROC curves for one-year OS; **(D)** ROC curves for threeyear OS; **(E)** ROC curves for five-year OS.

Clinical factors (including age, gender, grade, stage and risk score) were used to establish a novel nomogram for predicting one-year, three-year and five-year OS in smoking LSCC patients (Figure 6A). These results suggested that the advantage of a nomogram constructed using a combinatorial model is that it can better predict short- and long-term survival compared to a single prognostic factor. The novel nomogram might be helpful for clinical management of smoking LSCC patients. As shown in the time-dependent calibration curves (Figure 6B), there was a good agreement between the actual observation and the nomogram prediction. Also, the time-dependent ROC curves indicated that the nomogram was equipped with a high sensitivity and specificity in predicting OS of smoking LSCC patients (Figures 6C–E).

## Discussion

Laryngeal squamous cell carcinoma, a most common tumor of head and neck (Steuer et al., 2017), is prone to recurrence and metastasis (Obid et al., 2019). Patients who suffer from recurrent or metastatic LSCC and those with a poor response to platinum-based chemotherapy have a low survival rate (Saloura et al., 2014). Since the immune system plays a vital role in cancer development, immunotherapy is now extensively applied to counteract the immune escape against malignant cancer cells through regulating the key signaling pathways in the host immune system. In particular, cancer immunotherapy shows potentials of durable responses with fewer adverse effects than conventional treatments (Moy et al., 2017). The first cancer immunotherapy drug approved by the Food and Drug Administration (FDA) in 2011 was ipilimumab, a cytotoxic T-lymphocyte antigen 4 (CTLA4)-blocking monoclonal antibody (mAb) for metastatic melanoma.

Although the prognostic models of LSCC for predicting overall survival are constantly updated (Yang et al., 2016; Te Riele et al., 2018), immune-related prognostic index models of smoking LSCC patients have not been reported. In this study, we first identified differentially expressed IRGs of smoking LSCC patients, and the prognostic IRGs were subsequently screened out. Through establishing a prognostic index model, smoking LSCC patients were classified into the high-risk and the low-risk groups. Our findings demonstrated the great performance of the prognostic index model in predicting the prognosis of smoking LSCC patients as revealed by Kaplan-Meier and ROC curves.

To further explore the biological functions of IRGs in the development of LSCC, pathway enrichment analysis was conducted to depict the regulatory network. The KEGG analysis showed that prognostic IRGs were mainly enriched in cytokine-cytokine receptor interaction, natural killer cell mediated cytotoxicity and neuroactive ligand-receptor interaction. Immune cells and a network of pro-inflammatory and anti-inflammatory cytokines collaborate in cancer development and progression (Seruga et al., 2008). Cytokines are a heterogeneous group of soluble, small polypeptides or glycoproteins involved in virtually every aspect of immunity and inflammation (Borish and Steinke, 2003). It is believed that an environment rich in inflammatory cells, cytokines and activated stroma potentiates and/or promotes neoplastic risk (Balkwill and Mantovani, 2001).

In the constructed prognostic model, the following IRGs were subjected to the calculation of risk score: ULBP1, FAM19A4, EPO, TNFRSF25 and TUBB3. After reviewing the literature, we found that these IRGs may be closely related to immune response, but there were few reports about their relationship with head and neck cancer or laryngeal cancer.

As is well known, smoking not only increases the morbidity and mortality rates of cancer but also affects the immune system and functional immune activity (Shiels et al., 2014). In the analyses of immune contexts, we compared the infiltration of immune cells in different risk groups and found activated NK cells, M1 macrophages and resting mast cells was highly enriched in low-risk group. When it comes to functional immune activity, cytolytic activity and inflammation promoting were significantly activated in low-risk group. An obviously weaker immunoreactivity observed in high-risk group may explain the reason why patients in high-risk group had a poor outcome.

There are still some deficiencies in this study. First of all, we constructed a unique prognostic model for smoking LSCC patients by integrating IRGs expression based on TCGA database. However, in-depth analyses on clinical data from more independent cohorts are needed to confirm our findings. Secondly, we did not monitor the relative expressions of the selected five IRGs in smoking LSCC patients to assess their prognostic values. Thirdly, *in vivo* and *in vitro* functional experiments are needed to further validate our findings.

## Conclusion

We developed a novel IRGs-based prognostic index model for smoking LSCC patients, an interpretation of the mis-regulated tumor immune microenvironment. Also, these IRGs could be the potential therapeutic targets for smoking LSCC patients.

## Data Availability

The datasets analyzed during the current study are available in the TCGA repository (https://portal.gdc.cancer.gov) and GEO (https://www.ncbi.nlm.nih.gov/geo/).
