# Supplementary figures and images for: Smoking patients with laryngeal cancer screened with a novel immunogenomics-based prognostic signature
Source: Front Genet. 2022 Jul 14;13:961764. doi: 10.3389/fgene.2022.961764 (PMC9333188; doi:10.3389/fgene.2022.961764)

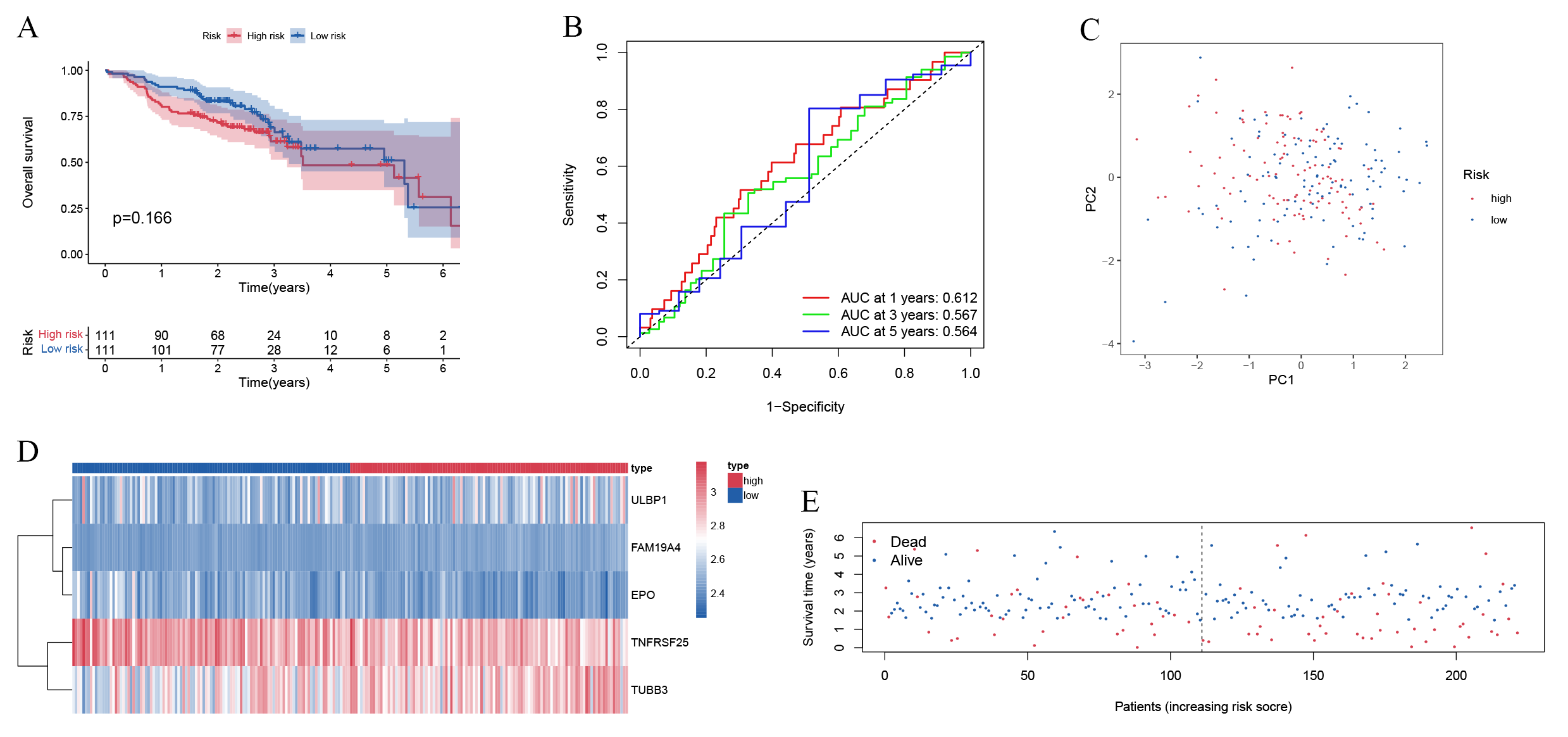

Supplement: Supplementary file 3 [file Image1.TIF]
